# The impact of the introduction of artificial intelligence in radiology and its potential legal implications in the UK and Ireland

**DOI:** 10.1259/bjro.20200030

**Published:** 2020-07-14

**Authors:** Toni Anderson, William C Torreggiani, Peter L Munk, Paul I Mallinson

**Affiliations:** 1 Tallaght University Hospital, Dublin, Ireland

## Abstract

Artificial intelligence (AI) has been defined as a branch of computer science dealing with the capability and simulation of a machine to imitate intelligent human behaviour. Diagnostic radiology, being a computer-based service, is unsurprisingly at the forefront of the discussion of the use of AI in medicine. There are however differing schools of thought regarding its use; namely, will AI eventually replace the radiologist? Or indeed will it ever be fully capable of replacing radiology as a speciality, but rather be used as an aid to the profession whereby a human’s input will always be required?

Furthermore, what will the legal implications of AI in radiology mean to the profession? Who will be liable for missed diagnoses? Is it possible that the introduction of AI to radiology will in fact make the profession busier?

## Introduction

Artificial intelligence (AI) has been defined as a branch of computer science dealing with the capability and simulation of a machine to imitate intelligent human behaviour. It was first portrayed in silent film in Fritz Lang’s Metropolis in 1927, and now AI is growing exponentially, stamping its mark on various industries, including of course, the medical profession.

In medicine, we have taken lead from the aviation sector with regard to their safety and communication protocols due to their relative success stories in learning from prior mistakes with a view to maximising “passenger,” or in our case, “patient” safety. In aviation, AI has had its role since the advent of the first basic autopilot in 1912. At present, the autopilot in an aeroplane has a high level of autonomy and the ability to retain information and make predictions, but ultimately the final control lies with the human pilot. In medicine, electrocardiograph machines can analyse a cardiac tracing and propose a differential diagnosis; however, it will always be examined by a doctor who will make the final determination.^1^

There are differing schools of thought regarding its use in diagnostic radiology; namely, will AI ever be fully capable of replacing radiology as a speciality? Or rather will it be used as an aid to the profession whereby a human’s input will always be required?

## The law of tort and medical negligence

In the legal world, a “tort” is defined as a civil wrong. Negligence is a branch of tort law whereby one party, usually although not always acting in a professional or contractual capacity, breaches a duty of care that is owed to another. For negligence to be established, one must be able to show that there was damage caused and also show that there was an element of “causation.” In other words, one must be able to link the action to the damage caused to the injured party. In medical terms, alleged acts of negligence manifest from the doctor–patient relationship, whereby the doctor has a professional and ethical duty of care to their patient. Medical negligence is the failure of a medical practitioner to provide proper care and attention that another similarly qualified practitioner would do in a similar circumstance. It is an act, or an omission to act, that is regarded as a deviation in accepted standards of practice that results in harm to the patient.^2^

The Bolam case,^3^ a landmark judgement in medical negligence law in the UK in 1957, recognised that a doctor is not immediately guilty of negligence where there is a poor patient outcome. This case highlights that a doctor will not be found negligent where it can be shown that they have acted in accordance with a practice that is widely accepted as reasonable by a group of doctors working in that same medical speciality. It also recognised that a doctor is not de facto negligent merely because there is a body of opinion which would adopt a different technique.

Indeed in a separate judgement in 1995, a surgeon was found not guilty of medical negligence in a case with a bad post-operative outcome, whereby he opted to use a less common choice of surgical procedure. This was notwithstanding the fact that 989 out of 1000 equally trained surgeons interviewed claimed that they would have opted for a different surgical approach. The court remarked that it was not for judges and lawyers to decide which medical and surgical approaches were reasonable and that 11 out of 1000 (1.1%) was enough to be considered *“a responsible body of medical males skilled in that particular art.”*

Similarly in Ireland, arguably the greatest medical negligence case to date was that of *Dunne vs National Maternity Hospital* in 1989.^4^ This landmark Supreme Court judgement laid out certain fundamental principles pertaining to medical negligence law, including but not limited to the concept that: *“the true test for establishing negligence in diagnosis or treatment on the part of a medical practitioner is whether he has been proved to be guilty of such failure as no medical practitioner of equal specialist or general status or skill would be guilty of it [when] acting with ordinary care.”* This essentially recognises that even when using the “ordinary level of care,” that doctors can, from time to time, get things wrong without being deemed negligent. Like Bolam, this case also recognises that there can reasonably be a *“honest different of opinion between doctors as to which is the better of two ways of treating a patient”* and that choosing treatment A over treatment B does not necessarily provide grounds for negligence.

## Litigation in radiology

AI has been flagged as a technology that could help to minimise the growing demands on radiologists. It is proposed that AI will first expediate diagnoses based on quick image interpretation.

The question then arises, whom is liable when software makes an error? To err is human, and as Garland pointed out in 1949,^5^ radiologists too are prone to human errors. As such radiologists can, and have been, held accountable for their actions.^6^ A review from the UK published earlier this year^7^ highlighted that almost a third of medicolegal claims over the last 11 years targeted radiology as the primary speciality at fault. Most of these cases involved an alleged missed or delayed diagnosis of a tumour on radiology imaging. An article published last year regarding to the use of robotics in healthcare demonstrated the current lack of liability framework where AI is concerned. The authors however went on to highlight that although liability regulations will be needed, it would be important not to allow these rules to be so stringent as to impede important new technological developments.^8^

If, for example, a software flaw is inherent in an image diagnostics programme, one might assume that the negligence liability will lie with the software developer who designed the AI package. The courts have however, to date, seemed reluctant to afford liability to the developer as in healthcare applications of AI. Instead, the final decision and hence liability has been deemed to rest with the radiologist.^9^

As things stand in diagnostic radiology, a clinical query is asked of the interpreting radiologist. Once the image is filmed, the onus is on the radiologist to report on the entire image, not solely on the organ system to which the initial clinical query pertains. The adage “two minds are better than one” comes to mind.

The question then arises, will AI’s high false-positive rate require the reporting radiologist to comment on every single highlighted aspect of a scan whether they think it pertinent or not? From a medicolegal point of view, it might be prudent of the radiologist to state *why* it is that they have ignored an alert deemed by the computer to be of significance.

It is proposed that in the absence of a CAD tool, should a radiologist omit to detect an early cancerous lesion, that it might be reasonable to infer that human error led to a missed diagnosis. This would be especially true if they could show that another radiologist *of equal status or skill could be guilty of such failure when acting with ordinary care*.

Conversely, it is proposed that if a radiologist using a CAD tool chooses to ignore a lesion highlighted by the software, and that lesion later turns out to be cancerous, the radiologist may not be deemed negligent if, at the time they have acknowledged the lesion in their official radiology report but, as in *Dunne vs National Maternity Hospital* expressed an *honest difference of opinion* with the software. Should they omit to do so however, it is suggested by this author that they will potentially face legal ramifications when a diagnosis is missed. I propose that in order to avoid liability, the radiologist will have to comment on each false-positive alert, thus massively increasing their workload.^10^ And furthermore could AI eventually be used to retrospectively prove negligence against the radiologist? Is it possible that the medicolegal fuel created by AI create a rise in cost in physician malpractice insurance thus leading ultimately to an increase in healthcare costs overall?

## Conclusion

Based on the literature, it is evident that not many foresee the imminent replacement of radiologists by AI. The common thought it that radiologists will remain a central and crucial cog in the diagnostic process of image-based medicine, with AI acting as a “cognitive companion.” It will likely improve patient outcomes and save money in the process. AI may in fact end up being implemented extensively first in jurisdictions where most radiographs are never reported.

However, if each alert by a diagnostics programme must be addressed individually and remarked upon, it could in fact mean an increased workload for the radiologist. This would be especially true in the early stages of AI in radiology and as such refute the idea that AI will replace radiology as a speciality as originally surmised.^10^
